# Use of Peer Groupings to Assess County Public Health Status

**Published:** 2008-06-15

**Authors:** Norma Kanarek, Ron Bialek, Jennifer Stanley

**Affiliations:** Johns Hopkins Bloomberg School of Public Health, Department of Environmental Health Sciences; Public Health Foundation, Washington, District of Columbia; Public Health Foundation, Washington, District of Columbia

## Abstract

**Introduction:**

The Community Health Status Indicators Project was undertaken to produce county-specific reports assessing the status of community health for local jurisdictions throughout the United States. To accomplish this assessment, the Community Health Status Indicators Project team selected peer groupings of counties to monitor and analyze the health of local communities relative to peer communities.

**Methods:**

To identify peer counties, the project team used 5 categorical county demographic variables, a specified order for applying criteria, and a predetermined target for peer grouping size to subdivide counties into homogeneous subgroups called peer groupings.

**Results:**

Eighty-eight peer groupings were developed with 14–58 counties in each. The average size of each peer grouping was 35 counties. All peer groupings included counties representing at least 6 states.

**Discussion:**

Peer groupings are very useful for community health assessment. They convey the range of health status indicator values for similar counties, serve as a basis for expected numbers of reportable diseases, and provide a method for comparing communities with peer and U.S. medians. To maintain their usefulness, peer groupings must be updated periodically.

## Introduction

Use of a comparison group to assess community health is as old as epidemiology itself and is the basis for calculating the expected values and relative risks of public health interventions and for assessing what levels of success those interventions are likely to achieve. The first step in prevention and control of chronic disease is to assess a community's standing with respect to chronic disease outcomes. Such an assessment can identify the community's needs and serve as the basis for gathering support for new or revitalized interventions to address those needs. The Community Health Status Indicators (CHSI) Project was undertaken to produce county-specific reports assessing community health status relative to peer counties across the United States.

The CHSI Project sought to identify the appropriate comparison population for assessing a county's standing with regard to the incidence of chronic diseases. To accomplish this, the CHSI Project needed to assign each county a set of peer counties (1). We used the Health Resources and Services Administration's Area Resource File (ARF) (2) to define county aggregates. ARF defines these aggregates as a borough, a parish, a city, the state of Alaska, or an otherwise defined "local" area. In this article, we use "county" interchangeably with these other local aggregations. The project identified peer groups from among like counties and county aggregates throughout the United States (3).

The CHSI Project is ongoing and issued its first set of peer counties in 2000 (CHSI 2000). An updated set of peer groupings was issued in September 2007 (CHSI 2007).

Previous efforts to implement the concept of peer communities have taken 1 of 3 approaches: 1) subjective selection of 2 or 3 counties without regard for actual jurisdictional characteristics, often made out of convenience (4); 2) selection of other counties in a defined geographic area (e.g., in the same state) (5,6); or 3) selection of mathematical neighbors, those determined to be the shortest statistical distance on the basis of a weighted summary of several variables (7,8). Peers selected out of convenience are frequently in the same state or taken from communities in a common project, such as the Big Cities Inventory (9). Peers from the state to which the county belongs are the usual choice for state health departments, their vital statistics departments, or statewide Web-based data warehouses and are an example of peers from the same geographical area. States like Maryland, for instance, report mortality rates for its 23 counties and the city of Baltimore only (6); other states’ counties are not reported. Mathematical neighbors are determined by a weighted combination of several variables. The weighted combinations having the smallest size (i.e., shortest distance) are identified as neighbors (i.e., peers). Thus, peers may be selected to be within a specified “distance” from the index county or to be of a specified number of one’s mathematically closest neighbors. This approach may be viewed as a “gold standard”; however, such models are used less often because either the calculations are not evident to the user or the variable(s) used are not those the user would choose (10).

Designated peer groupings allow explicit comparisons of counties and may include rankings and statistical testing of differences observed between the county and its peers. More often, however, comparisons are implicitly made by the reader. In such cases, an alphabetical listing of counties and their health indicators is provided with no attempt to indicate whether the number (e.g., a mortality rate) represents a county's "good" or "poor" health. In a survey of hospital-initiated assessments of local health, Fielding and colleagues noted that they looked for a designation of whether the community was better or worse, health-wise, than the chosen comparison group, but found that that information was rarely available (11).

The CHSI Project compared each county it assessed to its peers. During the development and evaluation phases of the project, user feedback consistently noted that the feature of peer counties from throughout the United States was a value-added utility of the CHSI Project county report. Thus, peer comparisons became a cornerstone of the CHSI Project reports. This paper will describe the strategy used to provide peer comparisons, address constraints in developing peer groupings in the CHSI Project, and detail the CHSI Project's peer-grouping algorithm. We discuss our experience in determining health status indicators for U.S. counties and make recommendations for future peer-grouping strategies.

## Methods

In 1998, the CHSI Project assembled an advisory panel of academic public health services researchers and local, state, and federal public health representatives who guided the development of the project's local county reports (1). Member representatives of the National Association of County and City Health Officials (NACCHO) and the Association of State and Territorial Health Officers met independently to poll their colleagues to obtain their recommendations for approaching peer comparisons and other report content. NACCHO had developed needs assessment tools (e.g., Mobilizing for Action through Planning and Partnerships [MAPP] [12] and Assessment Protocol for Excellence in Public Health [APEX-PH] [13]), which assist local health departments in establishing priorities based on local health information. Because of NACCHO's close involvement in the CHSI Project, the project (14) and MAPP (12) specified many of the same indicators. For example, CHSI 2000 provided indicators such as low birth weight percentage, all-cause mortality, county population characteristics and other measures that are suggested in the MAPP "core indicators list." The CHSI Project advisory group and staff determined the format, size, and range of the report contents.

It was determined early on in the project design that county-specific reports would be brief but would represent as broad a perspective on public health as space and data would allow. Ultimately, the report template was 16 pages, including title and back pages. Much background material was published in a companion document (14) so that readers interested in detail about methods, such as International Classification of Diseases codes for cause-specific mortality, could access this information.

Three goals guided the development of peer groupings. First, peer groupings needed to be transparent and immediately understandable in order to easily communicate a county's standing among its peers. A second goal for the CHSI Project reports was to explicitly compare counties with their peers. The third goal was to use the wisdom of practitioners and academicians to ground peer-grouping formation.

### Criteria for peer groupings

Peer groupings were constructed with the 3 goals in mind. The first goal in creating peer groupings was to make the groupings a manageable size, 30–40 counties each, so that all members of any peer grouping could be listed in the county report. Because the entire CHSI 2000 Report was only 16 half-pages long, we arbitrarily constrained space for listing peer counties to 1 page. Nevertheless, the CHSI Project provided a relatively large set of peers for each grouping. This approach enabled the listing of counties in the CHSI Project report on 1 page and resulted in peer groupings with more than a few peers.

A second goal for the CHSI Project reports was to explicitly compare counties with their peers. Thus, the reports incorporated calculations such as expected numbers of reportable diseases based on peer experience, the peer grouping range for other indicators, and a symbol as to whether the county's data for a specific indicator was above the median of its peers or at or below the median of its peers. Expected numbers were obtained by calculating a rate for the peer group as a whole and comparing it with each peer grouping member's population. For each indicator and peer grouping, the range of values for 80% of the counties was represented by the 10th and 90th percentile, which excluded the highest 10% and the lowest 10% of values exhibited by counties in the peer grouping. When a county's rate was worse than the peer grouping's median value, a magnifying glass was printed alongside the county rates. When a county's rate was better than or equal to the peer grouping's median value, an apple was printed.

A third goal in generating peer groupings was use of wisdom and conventions from public health practice. From the experience and advice of our advisors, 5 criteria for grouping counties as peers were obtained and then applied to the creation of peer groupings. Thus, because NACCHO, in its periodic survey of local health departments, used particular population size categories to describe a community served by a local health department (15), CHSI Project peer groupings included the same county population size categories of  ≥1,000,000; 500,000–999,999; 250,000–499,999; 100,000–249,999; 50,000–99,999; 25,000–49,999; and <25,000 (15). The second criterion adopted was community-level poverty, which was thought to represent a number of health issues — access to primary health care, having resources such as health insurance, and having a usual source of health care (16). Age (i.e., being older than 65 years or younger than 18 years), like poverty, is a common determinant of health services use and thus was included as another peer grouping criterion (17). Researchers and practitioners on the advisory group noted that the urban-rural continuum was a factor in relation to mortality and disease development (16); thus, frontier designation (population density <7 people per square mile) (18) and population density were deemed to be additional peer grouping variables (19).

### Selecting peer groups

The CHSI Project's steering committee provided guidance on the order in which these variables would be applied, based on their knowledge of how health services are organized (i.e., by frontier status, urban and rural population density factors) (20) and on informal use of population size (as in the Big Cities Inventory [9]) to designate peers. The criteria were applied in the following order: frontier status (yes/no), population size (7 categories), poverty (by quartile of U.S. counties), age distribution (deciles), and population density (half deciles). These criteria were applied until optimal size peer groupings were reached. At times only 2 criteria (nonfrontier status and population size) were used. Table 1 shows the 34 nonfrontier U.S. counties with populations of at least 1,000,000. In the case of this peer grouping, no further criteria were used.

The second and third peer groupings that CHSI 2000 created are distinguished from each other by poverty (the median proportion of all county populations living in poverty was ≤10.5%). Both peer groupings were made up of nonfrontier counties having a population size of 500,000–999,999, and included either low (≤10.5%) or high poverty levels (>10.5%). For most counties, all criteria were applied, and thus peer groupings were defined by frontier status, population size, poverty level, age distribution, and population density (18). Table 2 provides an example of a second peer grouping created using 4 criteria.

### Disease data aggregation

By design, the CHSI Project provided the most recent data to counties while assuring stable measures (i.e., sufficient sample size). Data were aggregated by peer grouping across 10, 5, or 3 years because peer groupings were made up of counties having similar population size. In summary, counties were subdivided into relatively homogeneous county subgroups using up to 5 categorical variables, a specified order for the criteria, and a predetermined target for peer grouping size.

## Results

From the 3082 counties in the United States, 88 peer groupings were designated, with sizes ranging from 14–58 counties and an average size of 35 (Table 3). One peer grouping comprised 14 counties, and 3 comprised 58 counties each. County peer groupings had low numbers or high numbers of counties when there were not enough counties in the predetermined categories, as was the case with frontier counties with populations >25,000, or when there were no criteria left by which to further divide subdivide the group.

Every peer grouping contained counties from multiple states (Table 4). Counties classified in a single peer grouping represented 6–25 states. Diversity of states in peer groupings was greatest in counties with populations of 100,000–249,999. In counties of this size, the modal number of states represented in peer groupings was 24. Peer groupings representing the smallest counties (<25,000 population) contained jurisdictions from fewer states; the modal number of states in peer groupings of counties with populations <25,000 was 11. No peer grouping had fewer than 6 states represented. Maps are another means of characterizing the diversity of states represented among peer groups (21).

Aggregation of data during a 3-, 5-, or 10-year period depended on the size of counties. Most counties (59%) were provided indicators aggregated during a 5-year period (Table 5), and nearly a quarter of counties (24%) were provided data in 10-year aggregates. Only about one-sixth of counties (17%) were eligible for 3-year rates because they had populations of ≥100,000.

### Use of peer groupings within the CHSI Project report

#### County and peer county demographics

The CHSI Project report provided population size and density, percentage of residents living in poverty, race/ethnicity, and age distributions. The report section presented the minimum and maximum values among the peers (1,14).

#### County status relative to the median

County health status indicator values were assessed as being above, equal to, or below the median value within the county's peer grouping. County indicators showing an outcome better than or equal to the median were noted with an apple symbol. Values for counties below the median were noted with a magnifying glass (1,14).

#### Peer groupings range

The range of values in a peer grouping was indicated by the 10th and 90th percentiles of county outcomes (1,14).

#### Peer grouping expected values

Peer counties' disease counts and populations were totaled and an overall rate generated for each peer group by dividing total cases by total population for the period. Expected number of cases for each disease (rounded to the nearest whole number) was obtained by multiplying the peer grouping rate by the county population (1).

#### Aggregation of data years

Indicators for natality and mortality were aggregated during varying numbers of years to balance the issues of using the most recent data available and providing an estimate that was relatively stable. The span of years presented is the same for all counties in any 1 peer grouping. Three-, 5-, and 10-year annual averages were calculated for populations ≥100,000, 25,000–99,999, and <25,000.

Indicators other than natality and mortality were presented throughout all counties only for a single year or 1 multiple-year period depending on the source of data involved. For example, toxic release substances were reported for 1 year while quality of life and life expectancy were reported for a single 5-year period. Suppression rules were applied to data to assure stability among the indicators presented (1,14).

## Discussion

CHSI 2000 incorporated peer groupings into the community health assessment assembled for each U.S. county, 3082 in all. Creation of peer groupings facilitated decisions about the number of data years to aggregate and allowed several states to be represented among a county's peers. Peer findings were integrated into the reports by indicating the number of cases of disease expected in a peer group, the range of the number of cases within the peer group, and whether a county was better than the median of its peers or of the United States.

The CHSI Project's approach to designating peers created 88 strata, based on the following hierarchically applied factors: frontier status, population size, poverty, age distribution, and population density. The approach yielded a peer grouping average size of 35 counties but did not avoid the creation of very small and very large peer groupings.

Diversity within peer groupings is greatest among those groupings of moderate size, no doubt because of the diversity that is present in states themselves. Few states have sparsely populated counties.

The peer grouping approach is readily transparent, easy to put into operation, and is consonant with local health departments, neighborhood planners, advocates, and citizenry who have an interest in local health (22). After we conducted this analysis, the Internet relaunch of the CHSI Project in June 2008 (CHSI 2008) used the same peer groupings but with the reassignment of Virginia cities and counties and the inclusion of Alaska as boroughs or counties. It is likely that counties will need to be reassigned in the future because of changes in their characteristics. A peer grouping should not be thought of as a static entity but rather as one in which members transition in and out because of changes in county population size and density, demographics, or boundaries. Even though membership in peer groupings will be updated, peer groupings remain a value-added characteristic of the CHSI Project report and a means for assessing just how well communities are faring relative to similar jurisdictions (1).

Practice-based alternative peer groupings should be examined with feedback from users of the CHSI Project reports. It may be that additional data necessary for determining new peer groupings are not yet available (e.g., county public health expenditures). In CHSI 2000, provision of county information immediately generated requests for neighborhood-level data and peers, data that are not available routinely yet. Sub-county areas such as neighborhoods may display the heterogeneity that is present in the county-level measures because counties may be quite large (22). Conversely, it is possible that counties identify with regions rather than with the entire country, illustrated by fewer states being represented in some of the peer groupings. Future iterations of the CHSI Project may allow the user an option of selecting a predefined peer grouping (e.g., multiple geographical groupings) or of specifying a peer grouping based on the user's own criteria.

Peer groupings have much utility for community health assessment, including conveying the range of health status indicator values for similar counties, a basis for expected numbers of reportable diseases, and a method for a median comparison. To maintain their utility, peer groupings must be updated periodically. Peer grouping criteria, such as population size and density and age composition, are components influencing county health outcomes (23) and will contribute to future research, particularly in the emerging field of public health services and public health services research (24). There are few examples of using like counties to benchmark a county's progress toward improvement in health outcomes, but CHSI 2000 was a first attempt to provide a comparison group of more than 2 or 3 peers for every U.S. county. In addition to being updated periodically, peer groupings may be continuously improved as our understanding of what constitutes a peer county grows and as the user community becomes more accomplished in using peers for comparison or benchmarking.

## Figures and Tables

**Table 1:** Members of One Community Health Status Indicator (CHSI) Project Peer Grouping Based on Nonfrontier^a^ Status and >1,000,000 Population, CHSI Project, 2000

Maricopa, Arizona Alameda, California Los Angeles, California Orange, California Riverside, California Sacramento, California San Bernardino, California San Diego, California Santa Clara, California Broward, Florida Dade, Florida Palm Beach, Florida	Cook, Illinois Middlesex, Massachusetts Oakland, Michigan Wayne, Michigan Hennepin, Minnesota St. Louis, Missouri Clark, Nevada Bronx, New York Kings, New York Nassau, New York New York, New York	Queens, New York Suffolk, New York Cuyahoga, Ohio Franklin, Ohio Allegheny, Pennsylvania Philadelphia, Pennsylvania Bexar, Texas Dallas, Texas Harris, Texas Tarrant, Texas King, Washington

a Nonfrontier counties have ≥7 people per square mile..

**Table 2:** Members of One Community Health Status Indicator (CHSI) Project Peer Grouping Based on Frontier Status^a^, <25,000 Population, High Poverty Level and Proportion of Children and Elders, and Low Population Density, CHSI Project, 2000

La Paz, Arizona Bent, Colorado Conejos, Colorado Costilla, Colorado Pondera, Montana Sanders, Montana McPherson, Nebraska Mora, New Mexico	Quay, New Mexico Union, New Mexico Benson, North Dakota Harmon, Oklahoma Roger Mills, Oklahoma Gregory, South Dakota Tripp, South Dakota	Briscoe, Texas Collingsworth, Texas Garza, Texas Jim Hogg, Texas Knox, Texas Presidio, Texas Terrell, Texas

a Frontier counties have a population density of <7 people per square mile.

**Table 3:** Distribution of Peer Groupings^a^ and Counties, by Frontier Status^b^, Population, and Poverty Prevalence, Community Health Status Indicators Project, 2000

Frontier Counties^a^				

Population Size	Poverty Prevalence			

	≤10.4 %	10.5%–14.1%	14.2%–19.0%	≥19.1%
<25,000: Groupings^b^ (no. of counties)	2 (69)	3 (130)	3 (101	3 (91)
≥25,000: Groupings (no. of counties)	1 (14)			

Nonfrontier Counties^a^				

Population Size	Poverty Prevalence			

	≤10.4 %	10.5–14.1%	14.2–19.0%	≥19.1%
<25,000: Groupings (no. of counties)	2 (56)	2 (108)	3 (142)	1(34)
25,000–49,999: Groupings (no. of counties)	9 (312)	7 (283)	10 (327)	16 (527)
50,000–99,999: Groupings (no. of counties)	4 (113)	3 (111)	3 (98)	1 (55)
100,000–249,999: Groupings (no. of counties)	3 (119)	3 (92)	1 (34)	1 (51)
250,000–499,999: Groupings (no. of counties)	2 (61)	1 (26)	1 (24)	
500,000–999,999: Groupings (no. of counties)	1 (31)	1 (39)		
≥1,000,000: Groupings (no. of counties)	1 (34)			

a Frontier counties have a population density of <7 people per square mile; nonfrontier counties have ≥7 people per square mile.

b There are a total of 88 peer groupings and 3082 counties. (Alaska is one county aggregate.)

**Table 4:** Distribution of States in Peer Groupings, by Population and County, Community Health Status Indicators Project 2000

Population	No. of Peer Groupings	Modal No. of States in Peer Grouping	Range
<25,000	19	11	7–22
25,000–49,999	43	11	6–25
50,000–99,999	9	18–19	9–22
100,000–249,999	7	24	12–24
≥250,000	10	18	14–23
Total	88	11	6–25

**Table 5:** Distribution of Years of Data Represented in Project Peer Groupings, by Population, Community Health Status Indicators Project, 2000

Population	No. of Counties	No. of Data Years Presented
<25,000	731 (24%)	10
25,000–49,999	1463 (47%)	5
50,000–99,999	377 (12%)	5
100,000–249,999	296 (10%)	3
≥250,000	215 (7%)	3
Total	3082 (100%)	3–10
