# Erratum, Vol. 5: Issue 3

**Published:** 2008-09-15

Because of an editing error, 2 references in the article "Use of Peer Groupings to Assess County Public Health Status" were cited incorrectly. The last paragraph of the "Discussion" should cite references 23 and 24, not 24 and 25. Corrections were made on our Web site on July 8, 2008, and appear online at www.cdc.gov/pcd/issues/2008/jul/07_0145.htm. We regret any confusion or inconvenience this error may have caused.

